# A retrospective review of α-gal syndrome complicating the management of suspected pancreatic exocrine insufficiency in one gastroenterology clinic in central Virginia

**DOI:** 10.3389/fgstr.2023.1162109

**Published:** 2023-07-26

**Authors:** Nathan E. Richards, Jeffrey M. Wilson, Thomas A. E. Platts-Mills, Robert D. Richards

**Affiliations:** ^1^ Division of Allergy and Clinical Immunology, University of Virginia, Charlottesville, VA, United States; ^2^ Gastroenterology Associates of Central Virginia, Lynchburg, VA, United States

**Keywords:** α-gal, α-gal syndrome, pancreatic exocrine insufficiency, pancreatic enzyme replacement, IgE, GI-variant α-gal syndrome, food allergy

## Abstract

The galactose-α-1,3-galactose (α-gal) mammalian meat allergy, α-gal syndrome, often includes diarrhea, abdominal pain, and other gastrointestinal (GI) symptoms. Pancreatic exocrine insufficiency causes similar symptoms. The pancreatic replacement enzymes, referred to here as pancreatic enzymes, used to treat pancreatic insufficiency are porcine products and contain α-gal. Patients with pancreatic insufficiency who also have α-gal syndrome may be intolerant of mammalian products in their diet and of α-gal in pancreatic enzymes. In this article, we describe 40 patients from one GI clinic in central Virginia with suspected pancreatic insufficiency and increased α-gal immunoglobulin E (IgE) levels. Over 50% of these patients had some clinical improvement when mammalian products were removed from the diet. Most patients could tolerate pancreatic enzymes; 10% could not tolerate them due to suspected allergy symptoms, but none developed anaphylaxis. Understanding that α-gal syndrome can be superimposed on pancreatic exocrine insufficiency and exacerbate symptoms, and that treatment with pancreatic enzymes may increase GI and/or allergy symptoms in this group, will lead to improved medical management of this complex patient population.

## Introduction

Immunoglobulin E (IgE) antibodies to the oligosaccharide galactose-α-1,3-galactose (α-gal) are an important cause of allergic reactions to mammalian meat and other mammal-derived products. The symptoms of α-gal syndrome can involve urticaria or anaphylaxis, but increasingly we are aware that gastrointestinal (GI) tract symptoms, including diarrhea, are also a major feature of α-gal syndrome (1–6). Pancreatic insufficiency is a common cause of diarrhea, and treatment involves the use of pancreatic enzymes, which are porcine derived and contain α-gal (7, 8). Patients receiving pancreatic enzymes who are α-gal IgE positive are at risk of allergic reactions and GI symptoms due to α-gal sensitivity (8, 9). There are limited clinical data available on this complex patient population, so we sought to address whether a mammalian product-restricted diet is helpful for these patients and also whether pancreatic enzymes are tolerated in α-gal-sensitized patients (10–12).

## Methods

A retrospective review of the electronic health records in one GI office, in an area of Central Virginia where α-gal syndrome is common, was carried out using inclusion criteria of (i) diarrhea, (ii) low levels of fecal elastase (< 200 μg/g feces), and (iii) α-gal IgE sensitization (> 0.10 kU/L). We began checking patients for α-gal IgE sensitization with increasing frequency in 2015, and the electronic health record query for this study was run in November 2022. Data collected from the electronic health record included sex, age, race, severity of diarrhea, allergy symptoms prior to starting pancreatic enzymes (including itch, hives, and angioedema), previous history of any pancreatic disease, pancreatic imaging done prior to starting pancreatic enzymes, any other previous or concomitantly diagnosed GI diseases, initial level of IgE to α-gal, initial level of fecal elastase, symptom response to removal of mammalian products from diet, if pancreatic enzymes had been prescribed and what type, and tolerance of pancreatic enzymes, including development of allergy symptoms or other GI symptoms. If subsequent repeat α-gal IgE titer was drawn, this was also recorded. As this was a retrospective review, there was not a standardized algorithm for working up diarrhea and this varied by practitioner and patient presentation, but, in general, α-gal level was drawn before or returned before fecal elastase and allowed for an initial trial taking patients off mammalian products.

Statistical analysis was performed using chi-squared testing to compare levels of IgE to α-gal with levels of fecal elastase between subgroups of this patient population.

## Results

Forty patients were identified, with an average age of 63.7 years (range 19–85 years), and 65% were female (see **Table 1** and the **Supplemental Data Table**). The median initial α-gal IgE level was 1.29 kU/L and the mean fecal elastase concentration was 137 μg/g. Pancreatic imaging included 31 CT scans, three MRIs, and one ultrasound. Twenty-six patients had normal pancreatic images, and nine had abnormal findings, including ductal abnormalities in three, atrophic findings in three, pancreatic cysts in two, fatty infiltration in two, and pancreatic mass in one. One patient had a history of hypertriglyceridemia-induced pancreatitis.

**Table 1:** Clinical data.

Characteristics	Total cohort (*n *= 40)
Age (years), mean (range)	63.7 (19–85)
Sex, female, *n* (%)	26 (65%)
Race, Caucasian, *n* (%)	37 (92.5%)
Race, African American, *n* (%)	3 (7.5%)
Diarrhea severity recorded, *n* (%)	26 (65%)
Mild, 0–4 stools per day, *n* (%)	12 (46%)
Moderate, 5–8 stools per day, *n* (%)	8 (31%)
Severe, > 8 stools per day, *n* (%)	6 (23%)
Improvement with mammalian-product avoidance, *n* (%)	21 (53%)
Treated with pancreatic enzymes, *n* (%)	30 (75%)
Creon, *n* (% of patients on pancreatic enzymes)	26 (87%)
Zenpep, *n* (% of patients on pancreatic enzymes)	3 (10%)
Pancreaze, *n* (% of patients on pancreatic enzymes)	1 (3%)
Total follow-up interval, months (range)	13 (2–59)

Eighteen of the 40 patients had other GI issues diagnosed concomitantly or pre-existing, which may have contributed to diarrhea, and were treated. These patients are listed in the **Supplemental Data Table**.

Of the 25 patients who had follow-up data available after eliminating mammalian-containing food products from the diet, 21 reported symptom improvement. With stopping mammalian products, six patients reported an improvement of their GI symptoms to the point that they did not require pancreatic enzymes.

Thirty patients were treated with pancreatic enzymes: 26 patients with Creon® (AbbVie Inc, Chicago, IL, USA), three with Zenpep® (Nestlé Health Science, Vevey, Switzerland), and one with Pancreaze® (Vivus LLC, Campbell, CA). Dosages of pancreatic enzymes were influenced by the provider’s prescription, medication cost, insurance coverage, and patient compliance. Unless noted in the **Supplementary Data Table**, the target pancreatic enzyme dosages were always at least 70,000 units of lipase per meal. Nine patients experienced symptoms thought to be related to pancreatic enzyme therapy: one with rash, three with itch, three with increased diarrhea, and two with nausea, vomiting, and bloating. Three of the patients stopped pancreatic enzymes because of the side effects. Of the 21 patients with follow-up information, 16 reported improvements while on treatment with pancreatic enzymes, and five reported no improvement.

Six of the 40 patients had pre-existing allergy symptoms of intermittent urticaria and/or pruritus before starting pancreatic enzymes that were attributed to α-gal syndrome. Levels of IgE to α-gal did not differentiate these patients. Of these six patients, one refused to try pancreatic enzymes due to the severity of their underlying symptoms. One patient with hives underwent office-based desensitization by his local allergist, starting with low-dose pancreatic enzymes and titrating the dose upward. One patient was not able to tolerate pancreatic enzymes due to an increase in diarrhea, and two patients were able to tolerate pancreatic enzymes (one of them with frequent pruritus). Finally, one patient’s symptoms resolved while avoiding mammalian products and did not require pancreatic enzyme therapy.

Levels of α-gal IgE were similar in those who responded and those who failed to respond to a mammal product-restricted diet, and also in those who tolerated or failed to tolerate pancreatic enzymes (**Table 2**). In addition, there was not a difference in levels of fecal elastase between those who responded and failed to respond to pancreatic enzymes.

**Table 2:** Clinical response relative to α-gal level and fecal elastase level.

	Median α-gal IgE level (kU/L)	*p*-value	Average fecal elastase level (μg/g)	*p*-value
Total patients, *n* = 40	1.29 (range 0.11–52.5)		137 (range 49–193)	
Clinical allergy symptoms prior to pancreatic replacement enzymes				
No symptoms, *n* = 34	1.15	0.509	132	0.204
Symptomatic, *n* = 6	1.33		156	
Response when off mammalian products (*n *= 25)				
Improved, *n* = 21	1.24	0.056	134	0.084
No improvement, *n* = 4	0.95		164	
Allergy symptoms on pancreatic replacement enzymes (*n *= 30)				
No symptoms, *n* = 21	1.34	0.405	138	0.839
Symptomatic, *n* = 9 (did not tolerate, *n* = 3; tolerated, *n* = 6)	0.57		130	
Improvement on pancreatic replacement enzymes (*n *= 21)				
Improved, *n* = 16	0.57	0.206	129	0.156
No improvement, *n* = 5	0.91		153	

In this review, 10 patients had a reduction in α-gal level over time (see the **Supplemental Data Table**). In one patient, the α-gal level decreased from 0.41 to < 0.10 kU/L over a time period of 2 years, and that patient was able to resume mammalian-product use. Three patients’ α-gal IgE levels increased.

## Discussion

This is the largest study to date looking at α-gal syndrome in a patient population with suspected pancreatic insufficiency requiring pancreatic enzymes. As all pancreatic replacement enzymes contain α-gal, both dietary recommendations and medical management may be influenced by α-gal allergy in this group (6, 7). Identifying α-gal syndrome as a potential contributing etiology in these patients will provide a clearer understanding of their underlying problems and better recommendations for management of their symptoms.

Alpha-gal syndrome is common in the southeastern United States and is diagnosed globally in many regions where ticks are endemic (12–16). GI symptoms are common in α-gal syndrome and may be the only presenting symptoms (2–4). We have previously identified that α-gal syndrome can be a sole cause of GI symptoms or can present superimposed on any pre-existing or concomitantly diagnosed GI disease (2). In this study, we performed a chart review of our GI office’s electronic health records to summarize our experience with patients who had laboratory assessments and symptoms suspicious for both α-gal syndrome and pancreatic insufficiency. This is a very complex patient population because they often present with multiple medical comorbidities, but even more so because it is so important to understand that diarrhea and GI symptoms in this group may be caused by so many intertwining etiologies. These include diarrhea and other GI symptoms related to pancreatic insufficiency, inadequate dosing of pancreatic enzymes, α-gal allergy related to mammalian-product consumption, α-gal allergy related to pancreatic enzyme product consumption, any other concomitant causes of diarrhea, or any combination of these.

Currently, a positive α-gal serology and response to a mammalian product-free diet are the important parts in the diagnostic paradigm for α-gal syndrome (17, 18). We and others have previously shown that patients with GI-variant α-gal syndrome have relatively low α-gal IgE levels compared with patients with α-gal syndrome presenting with classic allergy symptoms (2–4, 19). In this study, α-gal IgE levels could not be used to differentiate patients with prior systemic allergy symptoms from those without, those who would improve on pancreatic enzymes, or those who would develop allergy symptoms on pancreatic enzymes. Nevertheless, with 21 of 40 patients improving when taken off of mammalian products, and 9 of 30 patients developing classic allergy symptoms or increased GI symptoms while on pancreatic enzyme therapy, α-gal allergy is thought to be contributing to the pathogenesis of symptoms in this patient group. It is recognized that individuals may be sensitized to α-gal but nonetheless tolerate mammalian products, but there is not currently any test that can reliably distinguish between individuals who are symptomatic or asymptomatic (20–22). An oral food challenge requires significant resources and measures to mitigate risk, which are often not available. Well-designed placebo-controlled challenge studies may help further clarify the diagnostic markers of α-gal syndrome over time.

Alpha-gal syndrome is a dynamic allergy and not a static process, with α-gal IgE levels changing over time, as demonstrated in this study. In managing α-gal syndrome in the pancreatic insufficiency patient group, avoiding tick bite exposure is very important for long-term management. It is tick bite exposure that is thought to drive α-gal IgE levels up (12, 23–25). Avoiding mammalian-product exposure is the initial management plan for these patients and will help define α-gal syndrome as a contributing etiology. We currently do not have other proven treatments for α-gal syndrome (18, 26, 27). Understanding that all pancreatic enzymes currently contain α-gal is important because, if GI symptoms do not improve or are exacerbated with this treatment, it may not be because of patient non-compliance, but instead α-gal syndrome. Even though most of our patients were able to tolerate pancreatic enzymes, if patients have a history of urticaria or angioedema, we recommend anaphylaxis preparedness when initiating pancreatic enzyme therapy in α-gal-positive individuals. This would include patient education, availability of an epinephrine pen, and considering referral to an allergist. Finally, pigs have been developed that are α-gal free, so in the future there may be pancreatic enzymes and other porcine-derived medical products that are α-gal free (28).

This study is limited in several ways. It is a retrospective review of an office-based electronic health record and is not a prospective study that focused on complete capture of a predetermined data set, or strict treatment protocol for mammalian-product dietary exclusion, or predefined pancreatic enzyme dosing. During the chart review, we were generally able to separate symptom response to a mammalian product-free diet from response to pancreatic enzyme treatment, and from response to interventions for other GI issues based on the timing of these interventions and the clinical response. A prospective study could more clearly define these. Our diagnosis of suspected pancreatic insufficiency was based on GI symptoms, including diarrhea and a low fecal elastase. Fecal elastase is reported to be “the most sensitive and specific indirect test of pancreatic function,” and correlates well with tests of exocrine pancreatic function (29–31). Fecal elastase measurements can have false positives in patients with other non-pancreatic causes of malabsorption (32). Despite this limitation, fecal elastase is routinely used as the standard of care in clinical practice for diagnosis of pancreatic insufficiency. That three-quarters of the patients treated with pancreatic enzymes improved would suggest that pancreatic insufficiency was well represented in this study. The 17 other pre-existing or concomitantly diagnosed causes of diarrhea identified in these 40 patients increase the likelihood of false positives in the fecal elastase test and are reflections of how complex treating this patient population can be (32). Finally, this study was not placebo controlled for a mammalian product-avoidant diet or for pancreatic replacement enzyme therapy.

In conclusion, it is very important to recognize that α-gal syndrome can be superimposed on pancreatic insufficiency and exacerbate GI symptoms. Over half of these patients will experience symptomatic improvement by avoiding mammalian products. All pancreatic replacement enzymes currently contain α-gal and have the potential to cause allergy or GI symptoms in these patients. Worsening symptoms during pancreatic enzyme treatment may be caused by α-gal-related hypersensitivity rather than medication non-compliance. Nevertheless, most patients with α-gal sensitization and suspected pancreatic insufficiency could tolerate pancreatic enzymes in this study and none developed anaphylaxis. Recognition of α-gal syndrome superimposed on pancreatic insufficiency will allow improved management in this complex patient population.

## Data availability statement

The original contributions presented in the study are included in the article/**Supplementary Material**. Further inquiries can be directed to the corresponding author.

## Ethics statement

Ethics review and approval were not required for the study on human participants in accordance with the local legislation and institutional requirements. Written informed consent for participation was not required for this study in accordance with the national legislation and the institutional requirements.

## Author contributions

NR and RR carried out the chart review. All authors contributed to data analysis and interpretation. NR, RR, and JW contributed to the first draft of the manuscript. All authors provided critical feedback on the paper, had full access to the data, and accept responsibility for submission. All authors contributed to the article and approved the submitted version.

## Supplementary material

Supplementary Table Patient information.

## References

[1] Commins SP Satinover SM Hosen J Mozena J Platts-Mills TA. Delayed anaphylaxis, angioedema, or urticaria after consumption of red meat in patients with IgE antibodies specific for galactose-alpha-1,3-galactose. J Allergy Clin Immunol (2009) 123(2):426–33. doi: 10.1016/j.jaci.2008.10.052 PMC3324851 19070355

[2] Richards NE Richards RD. Alpha-gal allergy as a cause of intestinal symptoms in a gastroenterology community practice. South Med J (2021) 114(3):169–73. doi: 10.14423/SMJ.0000000000001223 33655311

[3] Richards NE Makin T Smith A Platts-Mills T Richards RD Wilson JM. The α-gal mammalian meat allergy as a cause of isolated gastrointestinal symptoms. Front Gastroenterol (2022) 1. doi: 10.3389/fgstr.2022.987713 PMC12952419 41822063

[4] Croglio MP Commins SP McGill SK. Isolated gastrointestinal alpha-gal meat allergy is a cause for gastrointestinal distress without anaphylaxis. Gastroenterology (2021) 160(6):2178–2180.e1. doi: 10.1053/j.gastro.2021.01.218 PMC9706491 33524403

[5] Mabelane T Basera W Botha M Thomas HF Ramjith J Levin M. Predictive values of alpha-gal IgE levels and alpha-gal IgE: total IgE ratio and oral food challenge-proven meat allergy in a population with a high prevalence of reported red meat allergy. Pediatr Allergy Immunol (2018) 29:841–9. doi: 10.1111/pai.12969 30144162

[6] Busing JD Stone CA Nicholson MR. Clinical presentation of alpha-gal syndrome in pediatric gastroenterology and response to mammalian dietary elimination. Am J Gastroenterol (2023) 118(7):293–1296. doi: 10.14309/ajg.0000000000002268 PMC10511000 36995329

[7] Swiontek K Morisset M Codreanu-Morel F Ollert M Eberlein B Hilger C. Drugs of porcine origin- a risk for patients with α-gal syndrome? J Allergy Clin Immunol: In Practice (2019) 7(5):1687–90. doi: 10.1016/j.jaip.2018.12.005 30557715

[8] Kuravi KV Sorrells LT Nellis JR Rahman F Walters AH Matheny RG. Allergic response to medical products in patients with alpha-gal syndrome. J Thorac Cardiovasc Surg (2022) 164(6):411–24. doi: 10.1016/j.jtcvs.2021.03.100 PMC9673037 33933257

[9] Stone CA Choudhary S Patterson MF Coleman DT Phillips EJ Commins SP. Tolerance of porcine pancreatic enzymes despite positive skin testing in alpha-gal allergy. J Allergy Clin Immunol: In Pract (2019) 8(5):1728–32. doi: 10.1016/j.jaip.2019.12.004 PMC7217730 31846796

[10] Eberlin B Mehlich J Reidenbach K Pilz C Hilger C Darsow U. Negative oral provocation test with porcine pancreatic enzyme plus cofactors despite confirmed alpha-gal syndrome. J Investig Allergol Clin Immunol (2020) 30(6):468–9. doi: 10.18176/jiaci.0513 32490824

[11] Molaei G Little EAH Williams SC Stafford KC. Bracing for the worst–range expansion of the lone star tick in the northeastern united states. N Engl J Med (2019) 381:2189–92. doi: 10.1056/NEJMp1911661 31800982

[12] Platts-Mills TAE Commins SP Biedermann T van Hage M Levin M Beck LA. On the cause and consequences of IgE to galactose-α-1,3-galactose: a report from the national institute of allergy and infectious disease workshop on understanding IgE-mediated mammalian meat allergy. J Allergy Clin Immunol Pract (2020) 145:1061–71. doi: 10.1016/j.jaci.2020.01.047 PMC7301618 32057766

[13] Fischer J Lupberger E Hebsaker J Blumenstock G Aichinger E Yadiz AS. Prevalence of type 1 sensitization to alpha-gal in forest service employes and hunters. Allergy (2017) 72:1540–7. doi: 10.1111/all.13156 28273338

[14] Hamsten C Tran TA Starkhammar M Commins SP Platts-Mills TAE van Hage M. Red meat allergy in Sweden: association with tick sensitization and b-negative blood groups. J Allergy Clin Immunol (2013) 132:1431. doi: 10.1016/j.jaci.2013.07.050 24094548 PMC4036066

[15] van Nunen SA O’Connor KS Clarke LR Boyle RX Fernando SL. An association between tick bite reactions and red meat allergy in humans. Med J Aust (2009) 190:510–11. doi: 10.5694/j.1326-5377.2009.tb02533.x 19413526

[16] Ebo DG Faber M Sabato V Leyson J Gadisseur A Bridts CH. Sensitization to the mammalian oligosaccharide galactose-alpha-1,3-galactose (alpha-gal): experience in a Flemish case series. Acta Clin Belg (2013) 68:206. doi: 10.2143/ACB.3278 24156221

[17] McGill SK Hashash JG Platts-Mills TAE. AGA clinical practice update on alpha-gal syndrome for the GI clinician: commentary. Clin Gastro Hep (2023) 21(4):891–6. doi: 10.1016/j.cgh.2022.12.035 36958889

[18] McGill SK Richards RD Commins SP. Suddenly steakless: a gastroenterologist's guide to managing alpha-gal allergy. Am J Gastro (2022) 117(6):822–6. doi: 10.14309/ajg.0000000000001765 35404302

[19] Liu K Wofford RN Newcomb DC Stone CA Moncayo A. Active surveillance of alpha-gal (galactose-alpha-1,3-galactose) syndrome in middle Tennessee. Ann Allergy Asthma Immunol (2023) 131(1):123–125. doi: 10.1016/j.anai.2023.04.005 37080457

[20] Roman-Carrasco P Hemmer W Cabezas-Cruz A Hodzic A de la Fuente J Swoboda I. The alpha-gal syndrome and potential mechanisms. Front Allergy (2021) 2:783279. doi: 10.3389/falgy.2021.783279 35386980 PMC8974695

[21] de la Fuente J Cabezas-Cruz A Pacheco I. Alpha-gal syndrome: challenges to understanding sensitization and clinical reactions to alpha-gal. Expert Rev Mol Diagn (2020) 20(9):905–11. doi: 10.1080/14737159.2020.1792781 32628573

[22] Mehlich J Fischer J Hilger C Swiontek K Morisset M Codreanu-Morel F. The basophil activation test differentiates between patients with alpha-gal syndrome and asymptomatic alpha-gal sensitization. J Allergy Clin Immunol (2019) 143:182–9. doi: 10.1016/j.jaci.2018.06.049 30125663

[23] Commins SP James HR Kelly LA Pochan SL Workman LJ Perzanowski MS. The relevance of tick bites to the production of the oligosaccharide alpha-gal. J Allergy Clin Immunol (2011) 127:1286–93. doi: 10.1016/j.jaci.2011.02.019 PMC3085643 21453959

[24] Steinke JW Platts-Mills TAE Commins SP. The alpha gal story: lessons learned from connecting the dots. J Allergy Clin Immunol (2015) 135:589–97. doi: 10.1016/j.jaci.2014.12.1947 PMC4600073 25747720

[25] Levin M Apostolovic D Biedermann T Commins SP Iweala OI Platts-Mills TAE. Galactose alpha-1,3-galactose phenotypes: lessons from various patient populations. Ann Allergy Asthma Immunol (2019) 122:598–602. doi: 10.1016/j.anai.2019.03.021 30922956 PMC6839685

[26] Commins SP. Omalizumab reduces food allergy symptoms in patients with alpha-gal syndrome. J Allergy Clin Immunol (2020) 145(2):AB145. doi: 10.1016/j.jaci.2019.12.468

[27] Bernal M Huecker M Shreffler J Mittel O Mittel J Soliman N. Successful treatment for alpha gal mammal product allergy using auricular acupuncture: a case series. Med Acupuncture Oct (2021) 33(5):343–348. doi: 10.1089/acu.2021.0010 PMC8729907 35003502

[28] Griffith BP Goerlich CE Singh AK Rothblatt M Lau CL Shah A. Genetically modified porcine-to-human cardiac xenotransplantation. N Engl J Med July (2022) 387:35–44. doi: 10.1056/NEJMoa2201422 PMC10361070 35731912

[29] Stevens T Conwell DL. Pancreatic exocrine insufficiency, in: UpToDate. Waltham, MA: UpToDate (Accessed December 3, 2022).

[30] Loser C Mollgaard A Folsch UR. Faecal elastase 1: a novel, highly sensitive, and specific tubeless pancreatic function test. Gut (1996) 39:580–6. doi: 10.1136/gut.39.4.580 PMC1383273 8944569

[31] Dominici R Franzini C. Faecal elastase 1 as a test for pancreatic function: a review. Clin Chem Lab Med. (2002) 40(4):325–32. doi: 10.1515/CCLM.2002.051 12059069

[32] Amann ST Bishop M Curington C Toskes PP. Fecal pancreatic elastase 1 is inaccurate in the diagnosis of chronic pancreatitis. Pancreas (1996) 13:226–30. doi: 10.1097/00006676-199610000-00002 8884841

