# Supplementary material for: A retrospective review of α-gal syndrome complicating the management of suspected pancreatic exocrine insufficiency in one gastroenterology clinic in central Virginia
Source: Front Gastroenterol (Lausanne). 2023 Jul 26;2:1162109. doi: 10.3389/fgstr.2023.1162109 (PMC12952332; doi:10.3389/fgstr.2023.1162109)
Supplement: Supplementary Table — Patient information. [file Table_1.docx]

**Supplemental Data Table.** Patient information

| **#** | **Age (Sex)** | **Diarrhea (episodes per day)** | **1st a-gal IgE kU/L** | **Repeat a-gal IgE kU/L (months)** | **Fecal Elastase** | **Pancreatic Imaging** | **Prior Allergy Symptoms: Pruritus** | **Prior Allergy Symptoms: Hives** | **Prior Allergy Symptoms: Angioedema** | **Diarrhea improved off a-gal** |
| --- | --- | --- | --- | --- | --- | --- | --- | --- | --- | --- |
| 1 | 52 (M) | Moderate | 5.46 | 4.92 (9) | 87 | Normal CT Pancreas |  |  |  | Resolved |
| 2 | 68 (M) | Mild (4) | 0.48 | 0.22 (5) | 49 | Normal CT Pancreas |  |  |  | Improved |
| 3 | 73 (F) | Yes | 0.57 |  | 128 | MRI: 8mm Uncinate cyst |  |  |  | No |
| 4 | 19 (M) | Moderate (7) | 6.37 | 0.61 (10) | 59 | Normal CT Pancreas |  | Yes |  | Improved |
| 5 | 80 (F) | Moderate (5) | 0.8 |  | 182 | CT: mild Pancreatic atrophy and fatty infiltrate |  | Yes |  | Resolved |
| 6 | 78 (M) | Mild (2) | 0.42 |  | 101 | Normal CT Pancreas |  |  |  | Improved |
| 7 | 63 (F) | Severe (12) | 1.24 |  | 163 | Normal CT Pancreas | Yes | Yes | Yes | Improved |
| 8 | 58 (F) | Moderate (6) | 0.91 |  | 176 | CT: mild Pancreatic duct dilation |  |  |  |  |
| 9 | 57 (F) | Mild | 0.43 |  | 148 | Normal CT Pancreas |  |  |  | Improved |
| 10 | 47 (F) | Moderate (8) | 0.96 |  | 103 | Normal CT Pancreas |  |  |  | Improved |
| 11 | 63 (M) | Mild | 0.41 | <0.10 (24) | 116 | MRI: 4mm IPMN cyst, prominent ductal side branches |  |  |  | Improved |
| 12 | 41 (M) | Mild (3) | 2.36 |  | 183 |  |  |  |  |  |
| 13 | 72 (F) | Moderate (7) | 6.89 |  | 177 | CT: moderate atrophy and fatty infiltrate |  | Yes |  |  |
| 14 | 70 (F) | Severe (12) | 1.25 |  | 175 | Normal CT Pancreas | Yes |  |  | Improved |
| 15 | 51 (F) | Severe (20) | 26.1 |  | 126 | Normal CT Pancreas |  |  |  | Improved |
| 16 | 57 (F) | Mild (3) | 10.4 | 5.79 (5) | 67 | Normal CT Pancreas |  |  |  | Resolved |
| 17 | 41 (M) | Mild (3) | 4.38 |  | 74 |  |  |  |  | Did not stop mammalian products |
| 18 | 76 (F) | Mild (4) | 0.15 |  | 121 | Normal CT Pancreas |  |  |  |  |
| 19 | 55 (F) | Moderate (8) | 0.34 |  | 146 | Normal CT Pancreas |  |  |  |  |
| 20 | 78 (F) | Mild (4) | 0.57 |  | 130 | Normal CT Pancreas |  |  |  | No, still eating dairy |
| 21 | 50 (F) | Moderate (7) | 1.91 | 0.38 (9) | 154 | Normal CT Pancreas |  |  |  | Resolved |
| 22 | 61 (F) | Mild (4) | 6.4 | 0.79 (16) | 121 | Normal CT Pancreas |  |  |  | No, still eating dairy |
| 23 | 79 (M) | Mild (3) | 8.56 |  | 104 |  |  |  |  |  |
| 24 | 70 (M) | Mild (4) | 1.34 |  | 178 | Normal CT Pancreas |  |  |  | No |
| 25 | 65 (F) | Yes | 0.15 |  | 167 |  |  |  |  | Resolved |
| 26 | 67 (F) | Yes | 2.14 | 7.41 (6) | 68 | Normal CT and MRI Pancreas |  |  |  |  |
| 27 | 81 (M) | Yes | 14.3 |  | 118 | CT: Pancreatic atrophy |  |  |  | No, did not stop mammalian products |
| 28 | 76 (F) | Yes | 0.4 |  | 181 |  |  |  |  | Resolved |
| 29 | 79 (F) | Yes | 0.11 |  | 167 |  |  |  |  |  |
| 30 | 53 (F) | Severe | 8.72 | 3.53 (6) | 120 | Normal CT Pancreas |  |  |  | Improved |
| 31 | 80 (F) | Severe | 6.14 |  | 159 | Normal CT Pancreas |  |  |  | Resolved |
| 32 | 54 (F) | Yes | 0.42 |  | 175 | Normal CT Pancreas |  |  |  | No |
| 33 | 72 (F) | Yes | 29.8 | 52.2 (10) | 185 | CT: minimal pancreatic duct prominence |  |  |  | Resolved |
| 34 | 32 (M) | Yes | 0.2 |  | 49 | US: Mass of pancreatic head |  |  |  |  |
| 35 | 64 (F) | Yes | 0.39 | 0.21 (7) | 177 | Normal CT Pancreas |  |  |  | Resolved |
| 36 | 70 (M) | Mild | 7.54 |  | 113 | Normal CT Pancreas |  |  |  | No, did not stop mammalian products |
| 37 | 63 (M) |  | 0.6 | 1.41 (4) | 178 | Normal CT Pancreas | Yes |  |  | No |
| 38 | 85 (M) | Severe | 8.4 | 5.8 (7) | 131 | Normal CT Pancreas |  |  |  | Improved |
| 39 | 68 (F) | Yes | 18.2 |  | 193 | Normal CT Pancreas |  |  |  |  |
| 40 | 83 (F) | Yes | 0.16 |  | 160 | Normal CT Pancreas |  |  |  | Improved |

**Supplemental Data Table Continued.** Patient information

| **#** | **Prior GI History** | **Concomitant GI Diagnoses** | **Prescribed Pancreatic Enzymes** | **Tolerated Pancreatic Enzymes** | **Increased Allergy Sx on Pancreatic Enzymes** | **Increased GI Sx on Pancreatic Enzymes** | **Diarrhea Improved on Pancreatic Enzymes** | **Allergist Recommendation** | **Comments** |
| --- | --- | --- | --- | --- | --- | --- | --- | --- | --- |
| 1 |  |  | Yes | Yes |  |  | Yes |  |  |
| 2 |  | Fructose Intolerance, SIBO | Creon |  | Pruritus |  | Yes |  | Fructose intolerance treated with dietary management, SIBO treated with antibiotics. |
| 3 | Lung cancer on Keytruda |  | Creon | Yes |  |  | Yes |  | Increased intraepithelial lymphocytes on colon biopsy, treated with Budesonide. |
| 4 |  |  | Creon | Yes |  |  | Yes | Office Based Desensitization | Lost to University Hospital follow-up. |
| 5 |  | Collagenous Colitis |  |  |  |  |  | Avoid mammalian products | Collagenous Colitis treated with Budesonide. |
| 6 |  |  | Creon | Yes |  |  |  |  |  |
| 7 | Near-total colectomy for polyposis |  | Creon | Yes | Pruritus |  | Yes | Avoid mammalian products | Given Epi-pen. |
| 8 | Gastric bypass | Anastomotic Gastric Ulcer | Creon |  |  |  | No |  |  |
| 9 |  |  | ZenPep | Yes |  |  | Yes | Office Based Oral Challenge | Fecal incontinence not improved with colestipol. |
| 10 | IBS, GERD |  | Creon | No | Rash |  |  |  |  |
| 11 |  |  | Creon | Yes |  |  | Yes |  | Follow up a-gal IgE level undetectable, pancreatic exocrine insufficiency eventually sole diagnosis. |
| 12 | GERD |  | Creon |  |  |  |  |  |  |
| 13 |  |  |  |  |  |  |  | Avoid mammalian products, Avoid PRE | Given Epi-pen. |
| 14 | Cirrhosis |  | Creon |  |  | Diarrhea |  |  |  |
| 15 |  |  |  | No |  |  |  |  |  |
| 16 | Gastric bypass | SIBO | Creon | Yes |  | Nausea, Emesis, Bloating | Yes |  | SIBO treated with antibiotics. |
| 17 |  |  | No |  |  |  |  | No dietary change |  |
| 18 |  |  | Creon |  |  |  |  |  |  |
| 19 |  |  | Creon, Pancreaze |  | Rash |  | Yes |  | Creon changed to Pancreaze due to rash. |
| 20 |  |  | Creon |  |  | Diarrhea | No |  |  |
| 21 |  | Lactose Intolerance | No |  |  |  |  |  | Lactose intolerance managed with diet and lactase enzyme. |
| 22 |  | Collagenous Colitis | Creon |  |  |  | No |  | Collagenous Colitis treated with Budesonide. |
| 23 |  |  |  |  |  |  |  |  |  |
| 24 |  |  | Creon |  |  |  | No |  |  |
| 25 |  |  |  |  |  |  |  |  |  |
| 26 |  | 6 cm adenomatous cecal polyp | Creon |  |  |  | Yes |  | Cecal polyp endoscopically removed at University Hospital. |
| 27 | Food allergy: positive milk, wheat, corn, peanut IgE |  | Creon |  |  |  | Yes |  | Patient refused to stop mammalian products after acupuncture and no longer identified dietary exacerbation. |
| 28 | Barrett’s Esophagus |  | Creon | No |  | Nausea |  |  |  |
| 29 | Hypertriglyceridemia Pancreatitis |  | Zenpep | Yes |  |  | Yes |  |  |
| 30 | GERD |  |  |  |  |  | Yes |  | Initial symptoms started after wasp sting. |
| 31 | Cirrhosis | Lymphocytic Colitis | Creon | Yes |  |  | Yes |  | Lymphocytic colitis treated with Budesonide. Developed progressive dementia 1 year later, taken off Creon and diarrhea treated with Imodium on inpatient chart review. |
| 32 | Para-esophageal Hiatal Hernia repair |  | Creon | Yes |  |  | Yes |  |  |
| 33 |  | Sucrose-Isomaltase Deficiency |  |  |  |  |  |  | Treated with oral enzyme therapy and diet. |
| 34 |  |  |  |  |  |  |  |  |  |
| 35 |  |  | Creon | Yes |  |  | Yes |  |  |
| 36 |  |  | Zenpep |  |  |  |  |  | Refused to stop mammalian products, treated with acupuncture, Zenpep was not continued long-term. |
| 37 |  |  | Zenpep | Yes |  |  | Yes | Positive beef IgE, reintroduced mammalian products |  |
| 38 |  |  | Creon |  |  |  |  |  |  |
| 39 | Cirrhosis | Lactose and Fructose Intolerance, Sucrose-Isomaltase Deficiency | Creon | Yes |  |  | Yes |  | Fructose Intolerance treated with diet. Sucrose Isomaltase and Lactose Intolerance treated with oral enzyme therapy. Gastric varices treated with coil embolization. |
| 40 |  |  | Creon |  |  | Diarrhea | No |  |  |
